# Synthesis of PP2A-Activating PF-543 Derivatives and Investigation of Their Inhibitory Effects on Pancreatic Cancer Cells

**DOI:** 10.3390/molecules27103346

**Published:** 2022-05-23

**Authors:** Su Bin Kim, Yoon Sin Oh, Kwang Joon Kim, Sung Woo Cho, Seung Ki Park, Dong Jae Baek, Eun-Young Park

**Affiliations:** 1 College of Pharmacy, Mokpo National University, Jeonnam 58554, Korea; rlatnqls0801@naver.com (S.B.K.); kjkim0901@mokpo.ac.kr (K.J.K.); 2 Department of Food and Nutrition, Eulji University, Seongnam 13135, Korea; ysoh@eulji.ac.kr; 3 Department of Bio and Chemical Industry, College of Engineering, The University of Suwon, Hwaseong 18323, Korea; powerfuley@daum.net (S.W.C.); skpark@suwon.ac.kr (S.K.P.)

**Keywords:** sphingosine kinase, PF-543, anticancer, inhibitor, protein phosphatase 2A

## Abstract

Sphingosine kinase (SK) is involved in the growth of cells, including cancer cells. However, which of its two isotypes—SK1 and SK2—is more favorable for cancer growth remains unclear. Although PF-543 strongly and selectively inhibits SK1, its anticancer effect is not high, and the underlying reason remains difficult to explain. We previously determined that the tail group of PF-543 is responsible for its low metabolic stability (MS). In this study, compounds containing aromatic or aliphatic tails in the triazole group were synthesized, and changes in the SK-inhibitory effect and anticancer activity of PF-543 were assessed using pancreatic cancer cells. The compounds with aliphatic tails showed high inhibitory effects on pancreatic cancer cells but slightly lower selectivity for SK1. A compound with an introduced aliphatic tail activated protein phosphatase 2A (PP2A), showing an effect similar to that of FTY720. Molecular docking analysis revealed that the PP2A-binding form of this newly synthesized compound was different from that noted in the case of FTY720. This compound also improved the MS of PF-543. These results indicate that the tail structure of PF-543 influences MS.

## 1. Introduction

**Figure 1:**
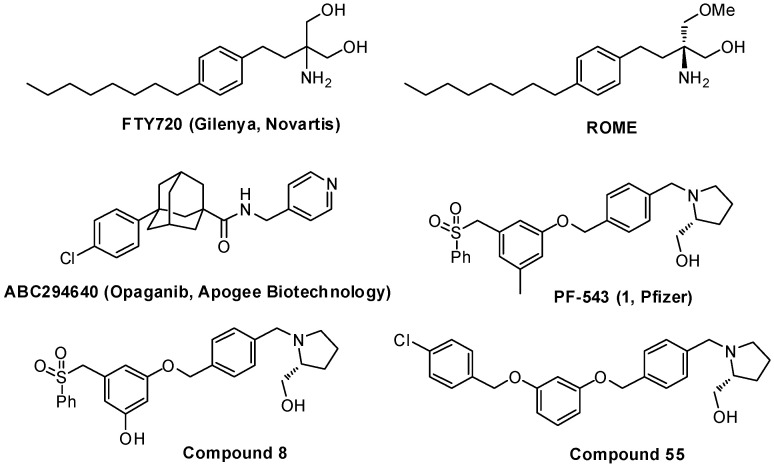
Known sphingosine kinase inhibitors and FTY720.

Sphingosine kinase (SK) induces cell growth by converting sphingosine to sphingosine-1-phosphate (S1P). S1P is associated with the growth of cancer cells, and its level has been shown to increase in patients with cancer [1]. Various biological studies have shown that the growth of cancer cells can be prevented by inhibiting SK. Therefore, several studies are currently focusing on the development of SK inhibitors and their application in various cancer therapies [2,3,4,5]. SK has two isotypes, SK1 and SK2 [5,6]. The growth of cancer cells has been reported to be suppressed when both the SK isotypes are inhibited. These isotypes are known to act by being located in different parts of the cell [7,8,9]. S1P generation, according to the location of SK1 and SK2, plays an important role in inducing the survival of cancer cells in specific oncogenic signaling pathways [10]. Various studies have attempted to control the selectivity of therapeutic compounds for SK1/2 through pharmacochemical methods; however, the correlation between a compound’s structure and its selectivity for SK1/2 remains obscure [11]. Only a small number of SK2-selective inhibitors have been developed thus far, and the crystal structure of SK2 has not been elucidated yet [2]. These factors pose significant challenges to the advancement of research in this domain. FTY720 is a therapeutic compound against multiple sclerosis. It was developed by Novartis, and it plays a role similar to that of sphingosine in vivo (Figure 1) [12]. FT720 acts as an S1P modulator, is transformed into FTY720-phosphate by SK2, and inhibits SK1. FTY720 exerts a strong anticancer effect by activating protein phosphatase 2A (PP2A). However, its activity is sensitive to even small structural changes in the head group. For example, ROME, which has a methoxy group introduced into FTY720, shows a change in activity and selectively inhibits SK2 (Figure 1) [13]. Previously developed SK inhibitors have structures similar to those of sphingosine and FTY720 [2]. Compounds with such long chains show poor solubility and stability in aqueous solutions [14]. Hence, pharmaceutical companies are currently developing nonlipid inhibitors of SK. For example, ABC294640 (Opaganib, Yeliva^®^ RedHill Biopharma), a nonlipid SK2 inhibitor, has passed phase 1 clinical trials for patients with various solid cancers and is currently under phase 2 clinical trials (Figure 1) [15]. PF-543, developed by Pfizer, inhibits SK1 more strongly than other nonlipid inhibitors of SK1 (Figure 1) [16]. PF-543 has shown efficacy against colorectal cancer in animal experiments, although in most studies using cancer cells, the anticancer effect was not as strong as the SK1-inhibitory effect [17]. The relatively low anticancer activity of PF-543 appears to be associated with the high sphingosine level compared with the low S1P level [2]. However, the correlation between the anticancer activity of PF-543 and changes in the levels of sphingosine and S1P has not been identified clearly. The results of studies on PF-543 analogs reported by Pfizer have suggested that PF-543 showed similar inhibitory effects to other analogs, and the head groups of PF-543 analogs do not significantly influence its selectivity for SK1/2 [18]. We have reported that compound **8**, in which the tolyl group backbone of PF-543 was modified with a phenol group (Figure 1), shows a reduced SK1-inhibitory effect [19]. This finding indicates that the tolyl or benzyl group of PF-543 should be maintained. In addition, derivatives modified with the tolyl group reportedly do not improve the low metabolic stability (MS) of PF-543. These results suggest that the tail of PF-543 is responsible for its low MS and also indicate the need for pharmacochemical modifications.

An in silico modeling study on the selectivity of PF-543 and SK1/2 demonstrated that PF-543 docked similarly onto SK1 and SK2, with only minute differences in the ligands and residues [20]. In a study reported by the Pyne group in 2019, the distance between the head and tail groups of PF-543 derivatives played an important role in the selectivity for SK1/2 [21]. In the case of compound **55** with a linker length longer than that of PF-543 reported in this paper (Figure 1), the IC_50_ of SK1 and SK2 were 4,130 and 41 nM, respectively, showing a sharp change in selectivity. These results show that a change in the tail structure is required to improve the activity of PF-543, which shows the possibility of developing analogs with selectivity for SK1/2 based on the structure of PF-543. We determined that the benzenesulfonyl group of PF-543 is responsible for its low MS and synthesized PF-543 analogs containing the triazole group [22,23]. Triazole is a known bioisostere of amides, is synthetically very convenient, and has a stable structure. To the triazole group, a phenyl tail such as that of PF-543 or an aliphatic tail such as that of FTY720 was added to compare the SK-inhibitory effect and assess the anticancer activity using pancreatic cancer cells.

## 2. Results and Discussion

### 2.1. Chemical Synthesis

**Scheme 1:**
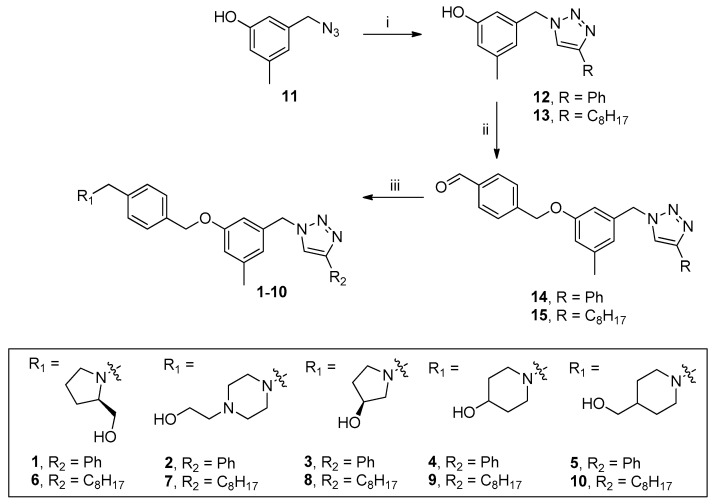
Synthesis of PF-543 derivatives (**1**–**10**). Reagents and conditions: (i) Na-ascorbate, phenylacetylene (for **12**) or 1-decyne (for **13**), CuSO_4_, *t*-BuOH/H_2_O (1/1), rt, 12 h; (ii) 4-(bromomethyl)benzaldehyde, K_2_CO_3_, THF, 50 °C, 12 h; (iii) cyclic amines, sodium triacetoxyborohydride (STB), 1,2-dichloroethane, rt, 12 h.

Compound **12** with a phenyl tail and compound **13** with an aliphatic tail were synthesized using compound **11** as a starting material by a click reaction and a known synthesis method [24]. Compounds **14** and **15**, into which an aldehyde was introduced, were synthesized through the reaction of compounds **12** and **13** with potassium carbonate (base), respectively. Final compounds **1**–**10** were synthesized through reductive amination using compounds **14** and **15** (Scheme 1).

### 2.2. Compounds with an Aliphatic Tail Structure Showed a Stronger Cytotoxic Effect Than Compounds with an Aromatic Tail Structure

**Figure 2:**
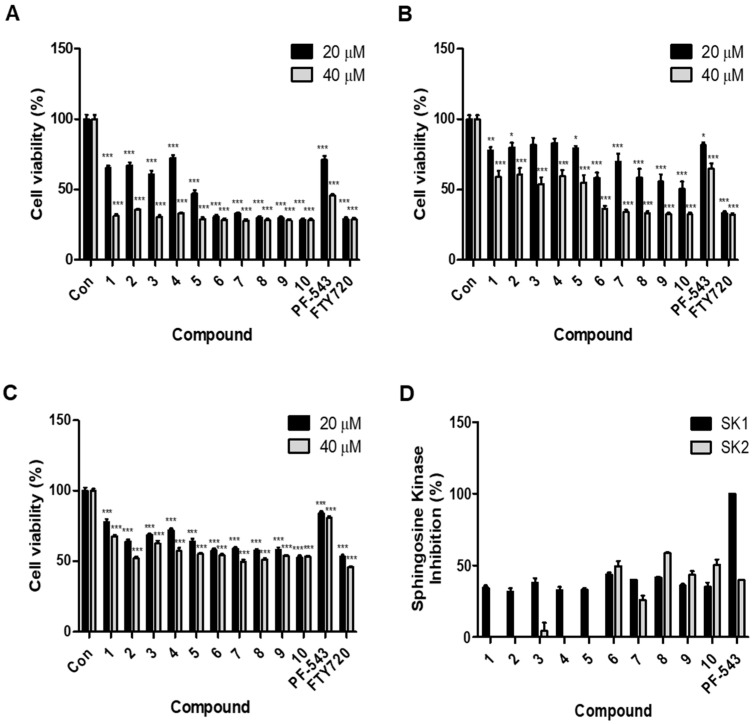
Cytotoxic effects of PF-543 and derivatives (**1**–**10**) in cancer cells. Cell viability was measured in (**A**) MIA PaCa2, (**B**) PANC-1, and (**C**) H6C7 cells treated with 20 and 40 µM PF-543 and derivatives. (**D**) Effects of PF-543 and derivatives (20 µM) on in vitro sphingosine kinase 1/2 assay. The experiments were carried out in triplicates and expressed as mean ± SD, one-way ANOVA, Turkey’s test. The values were considered significant at * *p* < 0.05, ** *p* < 0.01, and *** *p* < 0.001.

Using the pancreatic cancer cell lines MIA PaCa2 and PANC-1 and Human Pancreatic Duct Epithelial Cell Line H6C7, we measured the cytotoxic effects of the synthesized compounds **1**–**10**, PF-543, and FTY720 at 20 and 40 µM concentrations. All compounds showed a higher cytotoxic effect in the MIA PaCa2 cell line than in the PANC-1 cell line, and a significant cytotoxic effect was detected in both cell lines (Figure 2A,B). In both cell lines, compounds **1**–**5** with aromatic tail structures showed lower cytotoxic effects than compounds **6**–**10** with aliphatic tail structures. In both cell lines, compounds **1**–**5** showed a cytotoxic effect similar to that of PF-543, and compounds **6**–**10** showed a cytotoxic effect similar to that of FTY720. The IC_50_ values of compound **5**, compound **10**, and FTY720 were 26.07 μM, 11.14 μM, and 9.57 μM, respectively. The IC_50_ result shows that compound **10** exhibits a better cytotoxic effect than compound **5** (Supplementary Figure S1). The cytotoxic effect was less pronounced in Human Pancreatic Duct Epithelial Cell Line H6C7 (Figure 2C).

### 2.3. Compounds with an Aromatic Tail Structure Similar to PF-543 Have Selectivity for SK1 Inhibition

We measured the inhibitory effects of the synthesized compounds **1**–**10** on SK1 and SK2 using 20 µM concentrations of the compounds (Figure 2D). All compounds showed lower SK1-inhibitory effects than PF-543. Compounds **1**–**5** with aromatic tail structures similar to that of PF-543 showed selectivity for SK1 and inhibited this isotype. On the other hand, compounds **6**–**10** having an aliphatic tail did not show selectivity for SK1/2.

### 2.4. Compound ***10*** Effectively Reduces S1P and Sphingosine Levels

**Figure 3:**
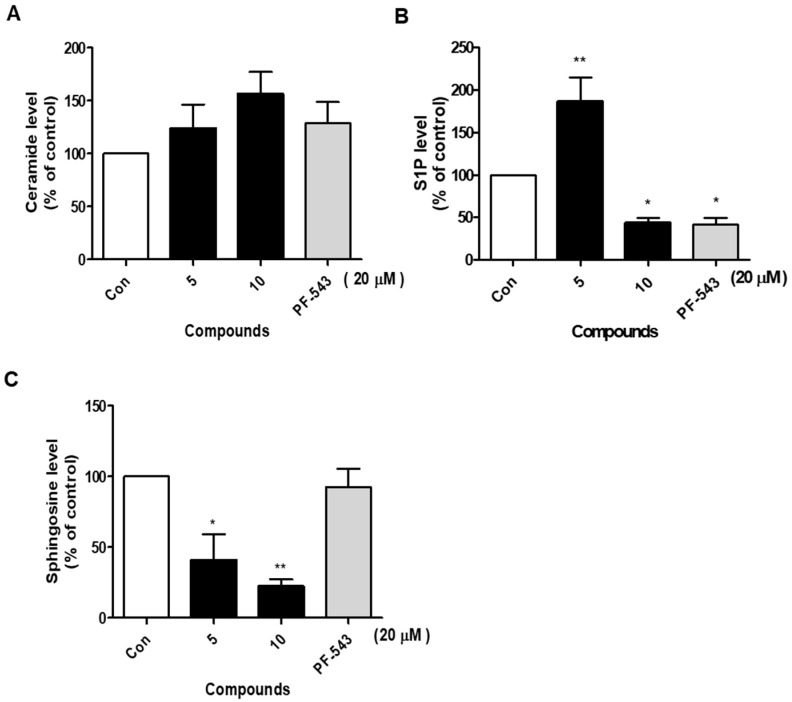
(**A**) Ceramide, (**B**) S1P, and (**C**) sphingosine levels were measured in MIA PaCa2 cells treated with compounds **5**, **10**, and PF543 at concentrations of 20 µM. The experiments were carried out in triplicates and expressed as mean ± SD, one-way ANOVA, Turkey’s test. The values were considered significant at * *p* < 0.05 and ** *p* < 0.01.

We measured the ceramide, S1P, and sphingosine levels of compound **5**, compound **10**, and PF-543 in pancreatic cancer cells using 20 µM concentrations (Figure 3). According to a previous study on PF-543, the ceramide level measured in cells derived from head and neck carcinoma specimens did not change based on the concentration of PF-543. However, because PF-543 lowered the S1P level based on its concentration, the sphingosine level increased [8]. In our study using pancreatic cancer cells, PF-543 did not significantly change the levels of ceramide and sphingosine but did reduce the level of S1P. This different result appears to be the cause of the low cytotoxic effect of PF-543 in pancreatic cancer cells. Compound **10**, which inhibited both SK1 and SK2, reduced the levels of S1P and sphingosine in pancreatic cancer cells. Compound **5** increased the S1P level, which was inconsistent with the direction of S1P reduction because of SK inhibition; this indicates the need for further studies regarding the influence of the compound’s structure on its SK-independent S1P-inhibitory effect.

### 2.5. Compound ***10*** Shows a Stronger Apoptosis Effect Compared to Compound ***5***

**Figure 4:**
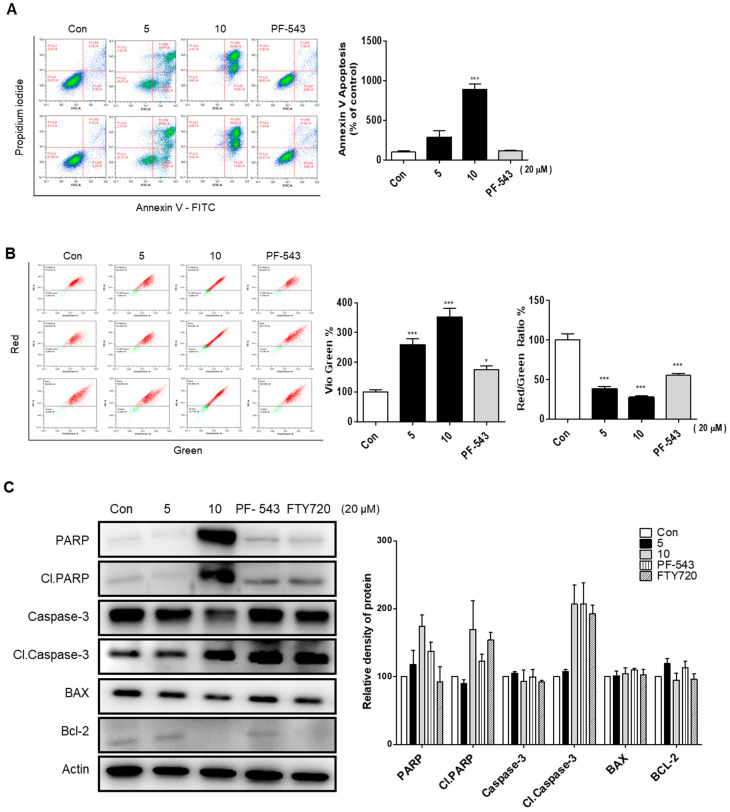
(**A**) MIA PaCa2 cell apoptosis effect was analyzed by annexin-V assay after treatment compounds **5**, **10**, and PF-543 (20 µM) using flow cytometry. (**B**) Compounds **5**, **10**, and PF-543 (20 µM) were treated in the MIA PaCa2 cell to measure JC-10 staining. (**C**) MIA PaCa2 cells were treated with compounds **5**, **10**, PF-543, and FTY720 (20 µM) for 24 h, and the expression of PARP, Cl.PARP, Caspase-3, Cl.Caspase-3, BAX, Bcl-2; Apoptosis markers. Actin was used as a control. The experiments were carried out in triplicates and expressed as mean ± SD, one-way ANOVA, Turkey’s test. The values were considered significant at * *p* < 0.05 and *** *p* < 0.001.

We performed annexin-V assays to evaluate the apoptotic effects of compound **5**, compound **10**, and PF-543 (Figure 4A). Unlike compound **5** and PF-543, compound **10** showed a high apoptotic effect, which is consistent with its cytotoxic effect noted in pancreatic cancer cells. This result might be because of the low S1P level due to the SK-inhibitory effect. When stress such as DNA damage is exerted, it disrupts the mitochondrial membrane’s ability to regulate potential and induces apoptosis. To examine the changes occurring in the mitochondria during the induction of apoptosis with compound **5**, compound **10**, and PF-543, an experiment was performed using the JC-10 dye (Figure 4B). Compounds **5**, **10**, and PF-543 all significantly reduced the mitochondrial membrane potential, calculated as a decrease in the ratio of red to green fluorescence. Western blot was performed to confirm the effect of compound **5**, compound **10**, PF-543, and FTY720 on apoptosis-related protein expression in pancreatic cancer cells (Figure 4C). As a result, in the compound **10** treatment group, the pro-apoptotic proteins cleaved form of PARP and caspase-3 increased, and the anti-apoptotic protein Bcl-2 decreased. The results of the compound **5** treatment group were insignificant compared to the compound **10** treatment group. The results were consistent with those of the annexin-V assay; thus, compound **10** was confirmed to exert a higher apoptotic effect than compound **5** and PF-543.

### 2.6. Compound ***10*** with an Aliphatic Tail Activates PP2A

**Figure 5:**
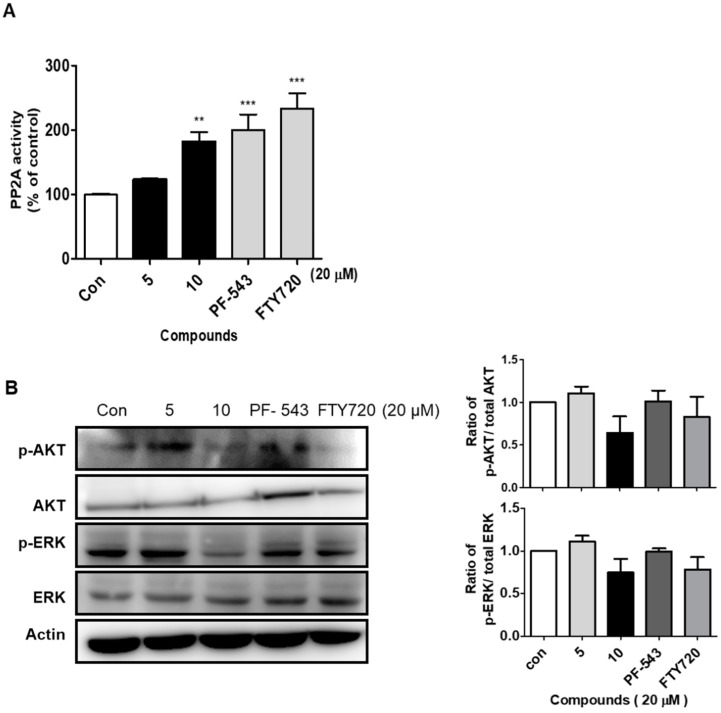
(**A**) PP2A activity of compounds **5**, **10**, PF-543, and FTY720 (20 µM) in MIA PaCa2 cells. (**B**) MIA PaCa2 cells were treated with compounds **5**, **10**, PF-543, and FTY720 (20 µM) for 24 h, then the expression of AKT, p-AKT, ERK, and p-ERK were probed by western blot. Actin was used as a control. The experiments were carried out in triplicates and expressed as mean ± SD, one-way ANOVA, Turkey’s test. The values were considered significant at ** *p* < *0*.01 and *** *p* < *0*.001.

Compound **10**, which showed a high apoptotic effect, has an aliphatic tail similar to that of FTY720; therefore, PP2A activation appeared to be possible. To confirm this, the effects of compound **5**, compound **10**, PF-543, and FTY720 on PP2A were evaluated using MIA PaCa2 cells (Figure 5). Compound **5** with an aromatic tail showed little effect on PP2A, but compound **10** with an aliphatic tail activated PP2A. Surprisingly, PF-543 with no aliphatic tail also activated PP2A in pancreatic cancer cells. After evaluating the effect of the compound on the phosphorylation of the PP2A subproteins AKT and ERK, we noted that phosphorylation of the two proteins was decreased as the PP2A activity increased following treatment with the compound. These results indicate that the anticancer activity of compound **10** in pancreatic cancer cells is associated not only with the inhibition of SK but also with the activity of PP2A according to its structural features. However, it is difficult to explain why PF-543 shows low anticancer activity despite activating PP2A in pancreatic cancer cells.

### 2.7. Compound ***10*** Shows Relatively Superior Metabolic Stability Compared to Compound ***5***

We reported the low MS of PF-543 in a previous study and that the tolyl group of PF-543 did not affect MS [19]. These results show that the low MS of PF-543 may be influenced by the benzenesulfonyl structure. The MS of compounds **5** and **10** was measured to determine if our synthesized triazole structure improved the structural stability of parent PF-543 (Table 1). The stability of compounds **5** and **10** measured in human, dog, rat, and mouse liver microsomes was improved relative to that of PF-543, and the structure of compound **10** with an aliphatic tail showed relatively superior stability to that of compound **5** with an aromatic tail. These results show that the tail structure of PF-543 is primarily responsible for its instability.

### 2.8. Molecular Modeling Studies

**Figure 6:**
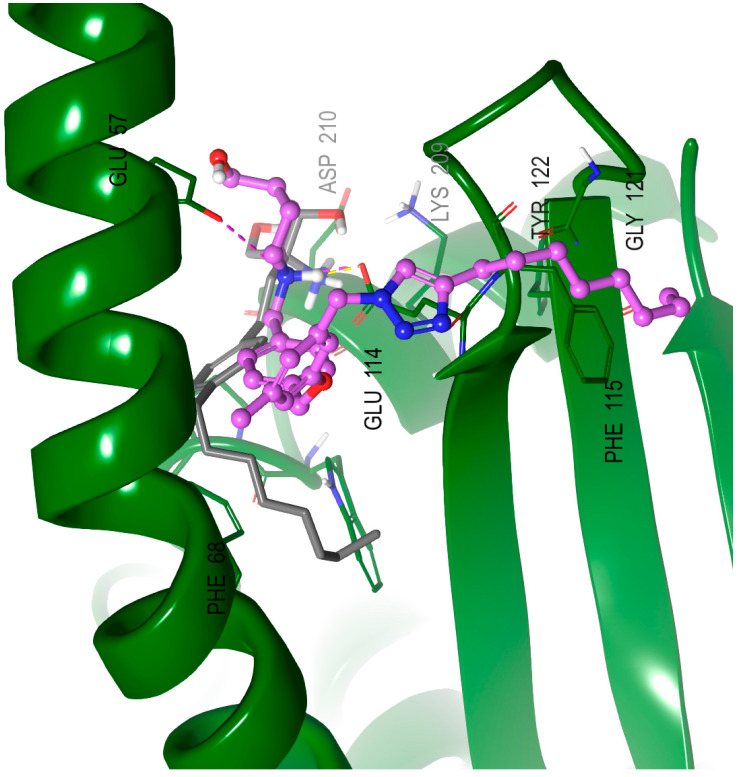
Docking model of FTY720 (gray, thick tube) and compound **10** (pink, ball and stick) in the I2PP2A/SET. The X-ray structure of IPP2A/SET was obtained from the Protein Data Bank (PDB code 2E50). The I2PP2A/SET is represented by a blue ribbon model. The hydrogen bonds are shown as a yellow dashed line, and a pink dashed line represents salt bridge interactions.

We performed molecular docking analysis to determine how compound **10** binds to PP2A (Figure 6). FTY720 and compound **10** were bound to PP2A at similar positions in the head group and backbone structure, but the triazole tail of compound **10** was bound to PP2A in a completely different direction from that noted in the case of FTY720. This might be because even if the compound has the same aliphatic tail structure, the tail structure can bind to PP2A in a completely different direction depending on whether the tail structure connected to the backbone is in a para form, as in the case of FTY720, or in a meta form, similar to that connected to the tolyl backbone. Our results show that even if the tail of the compound binds to PP2A in a direction different, it may lead to similar PP2A activity.

## 3. Experimental Section

### 3.1. Synthesis in General

The reagents used in the reaction were used by purchasing a commercially available reagent. Column chromatography was performed on silica gel grade 60 (230–400 mesh). All solvents used in the reaction were commercially available anhydrous solvents. ^1^H-NMR and ^13^C-NMR used JEOL ECZ500R (JEOL Co., Tokyo, Japan) and were measured using deuterated solvents at 500 and 125 MHz, respectively. High-resolution mass spectra were measured using an Agilent Technologies G6520A Q-TOF mass spectrometer (Santa Clara, CA, USA) instrument using electrospray ionization (ESI).

### 3.2. Chemical Synthesis

4-((3-Methyl-5-((4-phenyl-1*H*-1,2,3-triazol-1-yl)methyl)phenoxy)methyl)benzaldehyde (**14**), Compound **11** (0.54 g, 0.0033 mol) was dissolved in *t*-BuOH/H_2_O (1/1, 50 mL), and phenylacetylene (0.47 g, 0.0043 mol), Na-ascorbate (0.98 g, 0.0049 mol), and CuSO_4_ (0.78 g, 0.0049 mol) were added thereto. The mixture was stirred for 12 h at room temperature. The reaction was terminated with water and EtOAc. The phases were separated, the aqueous phase was extracted with EtOAc, organic extract was washed with brine, dried over MgSO_4_ and evaporated under reduced pressure. To a solution of crude compound **12** (0.0033 mol) mixture in THF (30 mL) were added K_2_CO_3_ (1.37 g, 0.0099 mol) and 4-(bromomethyl)benzaldehyde (0.79 g, 0.0040 mol). After the reaction mixture was heated at 50 °C for 12 h and the mixture was extracted with EtOAc. The combined organic phases were washed with brine, dried, and concentrated. Flash column chromatography with *n*-hexane:EtOAc (3:1 (*v/v*)) as the eluent gave **14** (0.67 g, 53%): ^1^H NMR (500 MHz, CDCl_3_) δ 9.93 (s, 1H), 7.84 (d, *J* = 8.2 Hz, 2H), 7.78 (dd, *J* = 8.3, 1.2 Hz, 2H), 7.63 (s, 1H), 7.53 (d, *J* = 8.1 Hz, 2H), 7.40 (t, *J* = 7.6 Hz, 2H), 7.33–7.30 (m, 1H), 6.77 (s, 1H), 6.74 (s, 1H), 6.65 (s, 1H), 5.48 (s, 2H), 5.09 (s, 2H), 2.31 (s, 3H); ^13^C NMR (125 MHz, CDCl_3_) δ 191.9, 159.0, 148.2, 143.7, 140.8, 136.1, 136.0, 130.4, 130.1 (2C), 129.0 (2C), 128.4, 127.6 (2C), 125.8 (2C), 121.8, 119.6, 116.2, 111.3, 69.2, 54.2, 21.6; ESI-HRMS (M + H)^+^ *m*/*z* calcd for C_24_H_22_N_3_O_2_ 384.1712, found 384.1748.

4-((3-Methyl-5-((4-octyl-1*H*-1,2,3-triazol-1-yl)methyl)phenoxy)methyl)benzaldehyde (**15**), Compound **11** (0.44 g, 0.0027 mol) was dissolved in *t*-BuOH/H_2_O (1/1, 50 mL), and 1-decyne (0.64 mL, 0.0035 mol), Na-ascorbate (0.81 g, 0.0041 mol), and CuSO_4_ (0.65 g, 0.0041 mol) were added thereto. The mixture was stirred for 12 h at room temperature. The reaction was terminated with water and EtOAc. The phases were separated, the aqueous phase was extracted with EtOAc, organic extract was washed with brine, dried over MgSO_4_ and evaporated under reduced pressure. To a solution of crude compound **13** (0.0027 mol) mixture in THF (30 mL) were added K_2_CO_3_ (1.12 g, 0.0081 mol) and 4-(bromomethyl)benzaldehyde (0.59 g, 0.0030 mol). After the reaction mixture was heated at 50 °C for 12 h and the mixture was extracted with EtOAc. The combined organic phases were washed with brine, dried, and concentrated. Flash column chromatography with *n*-hexane:EtOAc (3:1 (v/v)) as the eluent gave **15** (0.67 g, 59%): ^1^H NMR (500 MHz, CDCl_3_) δ 10.06 (s, 1H), 7.93 (d, *J* = 8.3 Hz, 2H), 7.61 (d, *J* = 8.1 Hz, 2H), 7.30 (s, 1H), 6.81 (s, 1H), 6.74 (s, 1H), 6.69 (s, 1H), 5.45 (s, 2H), 5.13 (s, 2H), 2.75–2.70 (m, 2H), 2.35 (s, 3H), 1.65–1.59 (m, 2H), 1.33–1.25 (m, 10H), 0.90 (t, *J* = 7.0 Hz, 3H); ^13^C NMR (125 MHz, CDCl_3_) δ 191.9, 158.9, 148.6, 143.7, 140.7, 136.3, 136.1, 130.1 (2C), 127.6 (2C), 121.8, 121.0, 115.9, 111.4, 69.2, 54.4, 31.9, 29.5, 29.4, 29.3, 29.2, 25.4, 22.7, 21.4, 14.2; ESI-HRMS (M + H)^+^ *m*/*z* calcd for C_26_H_34_N_3_O_2_ 420.2651, found 420.2636.

(*R*)-(1-(4-((3-Methyl-5-((4-phenyl-1*H*-1,2,3-triazol-1-yl)methyl)phenoxy)methyl)benzyl)pyrrolidin-2-yl)methanol (**1**), To a solution of **14** (40 mg, 0.1 mmol) and (*R*)-(-)-prolinol (32 mg, 0.31 mmol) in 1,2-dichloroethane (5 mL) was added sodium triacetoxyborohydride (STB) (44 mg, 0.21 mmol). After being stirred at rt for 12 h, the reaction mixture was diluted with water and extracted with EtOAc. The extract was washed with brine, dried, and evaporated. The residue was washed with CH_2_Cl_2_:MeOH (5:1 (*v*/*v*)) to give 39 mg (74%) of product **1**: ^1^H NMR (500 MHz, CDCl_3_) δ 7.79 (d, *J* = 7.1 Hz, 2H), 7.65 (s, 1H), 7.58 (d, *J* = 8.1 Hz, 2H), 7.42 (d, *J* = 8.3 Hz, 2H), 7.39 (d, *J* = 7.8 Hz, 2H), 7.34–7.29 (m, 1H), 6.76 (s, 1H), 6.73 (s, 1H), 6.62 (s, 1H), 5.47 (s, 2H), 5.01 (s, 2H), 4.31 (d, *J* = 13.1 Hz, 1H), 4.07 (d, *J* = 13.1 Hz, 1H), 3.81 (qd, *J* = 13.4, 4.8 Hz, 2H), 3.51 (dt, *J* = 8.9, 4.7 Hz, 2H), 2.89 (ddd, *J* = 11.4, 8.0, 6.9 Hz, 1H), 2.31 (s, 3H), 2.15–2.08 (m, 1H), 2.04–2.00 (m, 2H), 1.96–1.89 (m, 1H); ^13^C NMR (125 MHz, CDCl_3_) δ 159.1, 148.2, 140.7, 138.9, 136.1, 131.5 (2C), 130.6, 129.1, 129.0 (2C), 128.3, 128.1 (2C), 125.8 (2C), 121.7, 119.7, 116.2, 111.3, 69.3, 69.1, 60.9, 59.0, 54.2, 53.8, 26.4, 23.5, 21.6; ESI-HRMS (M + H)^+^ *m*/*z* calcd for C_29_H_33_N_4_O_2_ 469.2604, found 469.2691.

2-(4-(4-((3-Methyl-5-((4-phenyl-1*H*-1,2,3-triazol-1-yl)methyl)phenoxy)methyl)benzyl)piperazin-1-yl)ethanol (**2**), Compound **2** was prepared using 4-(2-hydroxyethyl)piperazine from compound **14** according to the same reaction procedure described for **1**: ^1^H NMR (500 MHz, CDCl_3_) δ 7.78 (d, *J* = 7.1 Hz, 2H), 7.64 (s, 1H), 7.42–7.37 (m, 2H), 7.33 (t, *J* = 9.4 Hz, 3H), 7.29 (d, *J* = 8.1 Hz, 2H), 6.77 (s, 1H), 6.72 (s, 1H), 6.66 (s, 1H), 5.47 (s, 2H), 4.98 (s, 2H), 3.87–3.83 (m, 2H), 3.69–3.62 (m, 2H), 3.56 (s, 2H), 2.93–2.89 (m, 2H), 2.79–2.73 (m, 4H), 2.62–2.50 (m, 2H), 2.31 (s, 3H); ^13^C NMR (125 MHz, CDCl_3_) δ 159.3, 148.3, 140.7, 136.3, 136.0, 132.1, 130.6, 129.7 (2C), 128.9 (2C), 128.3, 127.8 (2C), 125.8 (2C), 121.5, 119.6, 116.1, 111.4, 69.8, 61.8, 60.2, 59.5, 57.8, 56.8, 54.3, 52.9, 50.5, 21.6; ESI-HRMS (M + H)^+^ *m*/*z* calcd for C_30_H_36_N_5_O_2_ 498.2869, found 498.2821.

(*S*)-1-(4-((3-Methyl-5-((4-phenyl-1*H*-1,2,3-triazol-1-yl)methyl)phenoxy)methyl)benzyl)pyrrolidin-3-ol (**3**), Compound **3** was prepared using (*S*)-(-)-3-hydroxypyrrolidine from compound **14** according to the same reaction procedure described for **1**: ^1^H NMR (500 MHz, CDCl_3_) δ 7.77 (dd, *J* = 7.3, 1.0 Hz, 2H), 7.70 (s, 1H), 7.52 (d, *J* = 7.9 Hz, 2H), 7.43–7.36 (m, 4H), 7.34–7.29 (m, 1H), 6.76 (s, 1H), 6.73 (s,1H), 6.60 (s, 1H), 5.46 (s, 2H), 5.00 (s, 2H), 4.59–4.51 (m, 1H), 4.17 (d, *J* = 5.9 Hz, 2H), 3.48–3.43 (m, 1H), 3.30–3.14 (m, 3H), 2.30 (s, 3H), 2.29–2.21 (m, 1H), 2.13 (ddd, *J* = 12.7, 4.9, 2.9 Hz, 1H); ^13^C NMR (125 MHz, CDCl_3_) δ 159.3, 148.4, 141.0, 138.8, 136.2, 131.0 (2C), 130.7, 130.3, 129.2 (2C), 128.6, 128.4 (2C), 126.0 (2C), 121.9, 120.1, 116.4, 111.7, 69.8, 69.6, 61.1, 59.3, 54.5, 52.6, 33.9, 21.8; ESI-HRMS (M + H)^+^ *m*/*z* calcd for C_28_H_31_N_4_O_2_ 455.2447, found 455.2412.

1-(4-((3-Methyl-5-((4-phenyl-1*H*-1,2,3-triazol-1-yl)methyl)phenoxy)methyl)benzyl)piperidin-4-ol (**4**), Compound **4** was prepared using 4-hydroxypiperidine from compound **14** according to the same reaction procedure described for **1**: ^1^H NMR (500 MHz, CDCl_3_) δ 7.78 (d, *J* = 7.1 Hz, 2H), 7.67 (s, 1H), 7.51 (d, *J* = 8.0 Hz, 2H), 7.42–7.38 (m, 4H), 7.34–7.29 (m, 1H), 6.77 (s, 1H), 6.73 (s, 1H), 6.63 (s, 1H), 5.47 (s, 2H), 5.00 (s, 2H), 4.05–3.99 (m, 1H), 3.95 (s, 2H), 3.09 (t, *J* = 10.8 Hz, 2H), 2.92–2.89 (m, 2H), 2.31 (s, 3H), 2.28–2.23 (m, 2H), 1.87 (dd, *J* = 9.5, 4.3 Hz, 2H); ^13^C NMR (125 MHz, CDCl_3_) δ 159.4, 148.4, 141.0, 138.6, 136.2, 131.7, 130.7, 130.2, 129.2 (2C), 128.6 (2C), 128.3 (2C), 126.0 (2C), 121.9, 120.0, 116.4, 111.7, 69.7, 60.9, 56.7, 54.5 (2C), 50.5, 36.3, 30.9, 21.8; ESI-HRMS (M + H)^+^ *m*/*z* calcd for C_29_H_33_N_4_O_2_ 469.2604, found 469.2652.

(1-(4-((3-Methyl-5-((4-phenyl-1*H*-1,2,3-triazol-1-yl)methyl)phenoxy)methyl)benzyl)piperidin-4-yl)methanol (**5**), Compound **5** was prepared using 4-piperidinemethanol from compound **14** according to the same reaction procedure described for **1**: ^1^H NMR (500 MHz, CDCl_3_) δ 7.78 (d, *J* = 8.2 Hz, 2H), 7.67 (s, 1H), 7.53 (d, *J* = 8.0 Hz, 2H), 7.39 (dd, *J* = 8.1, 7.3 Hz, 4H), 7.33–7.29 (m, 1H), 6.76 (s, 1H), 6.72 (s, 1H), 6.63 (s, 1H), 5.47 (s, 2H), 5.00 (s, 2H), 4.00 (s, 2H), 3.51 (d, *J* = 6.2 Hz, 2H), 3.34 (d, *J* = 11.6 Hz, 2H), 2.63–2.51 (m, 2H), 2.30 (s, 3H), 1.88–1.77 (m, 4H); ^13^C NMR (125 MHz, CDCl_3_) δ 159.1, 148.2, 140.7, 138.5, 136.1, 131.6 (2C), 130.6, 129.4, 129.0 (2C), 128.3, 128.1 (2C), 125.8 (2C), 121.7, 119.7, 116.1, 111.4, 69.4, 66.0, 60.4, 54.2 (2C), 52.1, 31.0, 25.8 (2C), 21.6; ESI-HRMS (M + H)^+^ *m*/*z* calcd for C_30_H_35_N_4_O_2_ 483.2760, found 483.2782.

(*R*)-(1-(4-((3-Methyl-5-((4-octyl-1*H*-1,2,3-triazol-1-yl)methyl)phenoxy)methyl)benzyl)pyrrolidin-2-yl)methanol (**6**), Compound **6** was prepared using (*R*)-(-)-prolinol from compound **15** according to the same reaction procedure described for **1**: ^1^H NMR (500 MHz, CDCl_3_) δ 7.60 (d, *J* = 8.0 Hz, 2H), 7.44 (d, *J* = 8.0 Hz, 2H), 7.20 (s, 1H), 6.74 (s, 1H), 6.66 (s, 1H), 6.61 (s, 1H), 5.39 (s, 2H), 4.99 (s, 2H), 4.43 (d, *J* = 13.0 Hz, 1H), 4.17 (d, *J* = 13.1 Hz, 1H), 3.90–3.79 (m, 2H), 3.60–3.50 (m, 2H), 2.93 (dt, *J* = 11.4, 7.6 Hz, 1H), 2.70–2.62 (m, 2H), 2.29 (s, 3H), 2.06–1.78 (m, 4H), 1.70–1.65 (m, 2H), 1.66–1.55 (m, 2H), 1.33–1.19 (m, 8H), 0.84 (t, *J* = 7.0 Hz, 3H); ^13^C NMR (125 MHz, CDCl_3_) δ 159.1, 140.6, 138.9, 138.4, 136.4, 131.5 (2C), 128.1 (2C), 121.6, 117.8, 115.8, 113.7, 111.5, 69.3, 67.2, 62.8, 61.0, 53.9, 49.1, 31.9, 30.0, 29.5, 29.4, 26.4, 25.8, 24.4, 23.4, 22.7, 21.6, 14.2; ESI-HRMS (M + H)^+^ *m*/*z* calcd for C_31_H_45_N_4_O_2_ 505.3543, found 505.3529.

2-(4-(4-((3-Methyl-5-((4-octyl-1*H*-1,2,3-triazol-1-yl)methyl)phenoxy)methyl)benzyl)piperazin-1-yl)ethanol (**7**), Compound **7** was prepared using 4-(2-hydroxyethyl)piperazine from compound **15** according to the same reaction procedure described for **1**: ^1^H NMR (500 MHz, CDCl_3_) δ 7.35 (d, *J* = 8.1 Hz, 2H), 7.31 (d, *J* = 8.2 Hz, 2H), 7.18 (s, 1H), 6.75 (s, 1H), 6.66 (s, 1H), 6.62 (s, 1H), 5.39 (s, 2H), 4.96 (s, 2H), 3.91–3.86 (m, 2H), 3.63 (s, 2H), 3.22–3.02 (m, 4H), 3.01–2.97 (m, 2H), 2.88–2.72 (m, 4H), 2.69–2.63 (m, 2H), 2.29 (s, 3H), 1.65–1.57 (m, 2H), 1.25–1.22 (m, 10H), 0.85 (t, *J* = 7.0 Hz, 3H); ^13^C NMR (125 MHz, CDCl_3_) δ 159.3, 149.0, 140.5, 136.5, 136.3, 136.0, 129.7 (2C), 127.9 (2C), 121.5, 120.7, 115.8, 111.4, 69.7, 61.7, 60.1, 56.7, 54.0 (2C), 52.8, 50.2 (2C), 31.9, 29.5, 29.4 (2C), 29.3, 25.8, 22.7, 21.6, 14.2; ESI-HRMS (M + H)^+^ *m*/*z* calcd for C_32_H_48_N_5_O_2_ 534.3808, found 534.3833.

(*S*)-1-(4-((3-Methyl-5-((4-octyl-1*H*-1,2,3-triazol-1-yl)methyl)phenoxy)methyl)benzyl)pyrrolidin-3-ol (**8**), Compound **8** was prepared using (*S*)-(-)-3-hydroxypyrrolidine from compound **15** according to the same reaction procedure described for **1**: ^1^H NMR (500 MHz, CDCl_3_) δ 7.56 (d, *J* = 8.1 Hz, 2H), 7.41 (d, *J* = 8.0 Hz, 2H), 7.22 (s, 1H), 6.74 (s, 1H), 6.66 (s, 1H), 6.56 (s, 1H), 5.37 (s, 2H), 5.00 (s, 2H), 3.46 (s, 2H), 3.32–3.19 (m, 1H), 2.69–2.60 (m, 2H), 2.34–2.25 (m, 1H), 2.29 (s, 3H), 2.18–2.09 (m, 1H), 2.04–1.94 (m, 4H), 1.66–1.58 (m, 2H), 1.35–1.17 (m, 10H), 0.85 (t, *J* = 6.9 Hz, 3H); ^13^C NMR (125 MHz, CDCl_3_) δ 159.0, 148.9, 140.6, 138.6, 136.3, 130.8 (2C), 130.2, 128.1 (2C), 121.6, 120.9, 116.0, 111.5, 69.8, 69.6, 69.4, 65.5, 59.2, 54.0, 33.6, 31.9, 29.4 (2C), 29.3 (2C), 25.7, 22.7, 21.6, 14.2; ESI-HRMS (M + H)^+^ *m*/*z* calcd for C_30_H_43_N_4_O_2_ 491.3386, found 491.3332.

1-(4-((3-Methyl-5-((4-octyl-1*H*-1,2,3-triazol-1-yl)methyl)phenoxy)methyl)benzyl)piperidin-4-ol (**9**), Compound **9** was prepared using 4-hydroxypiperidine from compound **15** according to the same reaction procedure described for **1**: ^1^H NMR (500 MHz, CDCl_3_) δ 7.53 (d, *J* = 7.9 Hz, 2H), 7.42 (d, *J* = 8.0 Hz, 2H), 7.21 (s, 1H), 6.75 (s, 1H), 6.67 (s, 1H), 6.62 (s, 1H), 5.39 (s, 2H), 4.99 (s, 2H), 4.03 (s, 2H), 3.51–3.43 (m, 1H), 3.13 (dd, *J* = 14.4, 7.1 Hz, 2H), 3.01–2.85 (m, 2H), 2.69–2.63 (m, 2H), 2.30 (s, 3H), 2.27–2.17 (m, 2H), 1.88 (dd, *J* = 9.3, 3.9 Hz, 2H), 1.61 (dd, *J* = 14.4, 7.1 Hz, 2H), 1.38–1.17 (m, 10H), 0.85 (t, *J* = 6.8 Hz, 3H); ^13^C NMR (125 MHz, CDCl_3_) δ 159.1, 149.0, 140.6, 138.3, 137.0, 136.3, 131.4 (2C), 128.0 (2C), 121.6, 120.8, 115.8, 111.5, 69.5, 60.7, 54.0 (2C), 50.8, 45.5, 31.9 (2C), 30.8, 29.4 (2C), 29.3, 29.2, 25.7, 22.7, 21.6, 14.2; ESI-HRMS (M + H)^+^ *m*/*z* calcd for C_31_H_45_N_4_O_2_ 505.3543, found 505.3572.

(1-(4-((3-Methyl-5-((4-octyl-1*H*-1,2,3-triazol-1-yl)methyl)phenoxy)methyl)benzyl)piperidin-4-yl)methanol (**10**), Compound **10** was prepared using 4-piperidinemethanol from compound **15** according to the same reaction procedure described for **1**: ^1^H NMR (500 MHz, CDCl_3_) δ 7.47 (d, *J* = 8.0 Hz, 2H), 7.40 (d, *J* = 8.1 Hz, 2H), 7.19 (s, 1H), 6.75 (s, 1H), 6.67 (s, 1H), 6.63 (s, 1H), 5.39 (s, 2H), 4.99 (s, 2H), 3.90 (s, 2H), 3.25 (d, *J* = 11.7 Hz, 2H), 2.70–2.64 (m, 2H), 2.39 (t, *J* = 11.7 Hz, 2H), 2.30 (s, 3H), 2.09–1.97 (m, 2H), 1.83 (d, *J* = 12.6 Hz, 2H), 1.69 (dd, *J* = 19.1, 7.7 Hz, 2H), 1.66–1.58 (m, 3H), 1.36–1.17 (m, 10H), 0.85 (t, *J* = 7.0 Hz, 3H); ^13^C NMR (125 MHz, CDCl_3_) δ 159.2, 149.0, 140.6, 137.6, 136.4, 132.4, 131.0 (2C), 127.9 (2C), 121.5, 120.7, 115.8, 111.5, 69.6, 66.7, 61.2, 54.0 (2C), 52.3, 37.2, 31.9 (2C), 29.5, 29.4, 29.3 (2C), 26.7, 25.8, 22.7, 21.6, 14.2; ESI-HRMS (M + H)^+^ *m*/*z* calcd for C_32_H_47_N_4_O_2_ 519.3699, found 519.3615.

### 3.3. Chemicals and Reagents

Cell culture media DMEM and RPMI, trypsin-EDTA 0.25%, and penicillin–streptomycin were purchased from GE Healthcare Life Sciences Hyclone Laboratories (Pittsburg, PA, USA). Fetal bovine serum (FBS) was received from Gibco (Thermo Fisher Scientific, Inc., Walthem, MA, USA). Apoptosis detection kit, ApoScanTM annexin V-FITC was obtained from BioBud (Cat. No.: LS-02-100, Gyeonggi-do, Korea). MTT cell viability assay kit EX-CYTOX was acquired from Do-GenBio Co. Ltd. 9, Seoul, Korea). PP2A activity kit was purchased from Millipore Corporation (Billerica, MA, USA). Antibodies against caspase-3, BAX, PARP, AKT, ERK, p-AKT, and p-ERK were bought from Cell Signaling Technology (Danvers, MA, USA), antibodies against β-actin, Bcl-2, HRP-conjugated anti-mouse and anti-rabbit antibodies were received from Santa Cruz Biotechnology (Dallas, TX, USA), and antibodies against SK1 and SK1 were acquired from Abcam (Cambridge, UK). Protein marker, protease, and phosphatase inhibitor cocktail were purchased from Thermo Fisher Scientific (Waltham, MA, USA), and ECL Solution for Western blotting imaging was received from Millipore Corporation 9, Burlington, MA, USA). ELISA kit for ceramide, S1P, and sphingosine were acquired from MyBiosourse, Inc., (San Diego, CA, USA).

### 3.4. Sphingosine Kinase Activity Assay

SK 1/2 activity was measured using 20 μM PF-543 and compounds **1**–**10** using the AdaptaTM screening system (Thermo Fisher Scientific system). Sphingosine kinase 1 activity assay used 0.04–0.16 ng SPHK1, 50 μM sphingosine lipid substrate in 32.5 mM HEPES pH 7.5, 0.005% BRIJ 35, 5 mM MgCl_2_, 0.5 mM EGTA. Sphingosine kinase 2 activity assay detected 35–140 ng SPHK2, 50 μM sphingosine lipid substrate in 32.5 mM HEPES pH 7.5, 0.5 mM EGTA, and 1.5 mM MgCl_2_.

### 3.5. Cell Culture and Proliferation Assays

The human pancreatic ductal epithelial cell lines MIA Paca2, PANC-1 were maintained in DMEM media with 10% fetal bovine serum (FBS), 100 U/mL penicillin, and 100 µg/mL streptomycin at 37 °C in a humidified 5% CO_2_/95% air atmosphere. Cells were seeded in 96-well plates at a density of 3000 cells/100 μL/well and incubated for 24 h. The cells were then incubated in a culture medium containing synthetic compounds. Following 24 h of incubation, the cell viability was determined using an EZ-CYTOX kit (DaeilLab Service, Seoul, Korea) according to the manufacturer’s protocol (n = 12).

### 3.6. Ceramide, Sphingosine, S1P Level

Ceramide, sphingosine, and S1P level were performed as protocols recommended by Mybiosource using the ceramide, sphingosine, and S1P ELISA kit (MyBiosource Inc., San Diego, CA, USA). Ceramide and sphingosine S1P measurements were tested with proteins that were centrifuged and collected by repeating freezing and thawing from three to five times after adding PBS to cells.

### 3.7. Annexin-V Staining of PF-543, Derivative ***5***, and ***10***

Cell apoptosis assay was determined using annexin V- FITC and propidium iodide (PI) staining, as described in manufacturer instruction (BioBud, Gyeonggi-do, Korea). Briefly, the cells were resuspended in binding buffer and then incubated with annexin-V-FITC and propidium iodide at room temperature. After incubation, stained cells were analyzed by MACSQuant Analyzer 10 Flow Cytometer (Miltenyi Biotec, Bergisch Gladbach, Germany). The experiment was conducted three times independently (n = 6).

### 3.8. Mitochondrial Membrane Potential (MMP, Δψm)

To measure the degree of MMP change of cancer cells, 20 μM JC-10 dye and assay buffer (Enzo Co., New York, NY, USA) were added to the cells cultured for 24 h and mixed until the cells were completely dissolved. Subsequently, the cells were incubated in the dark for 30 min at room temperature. The solutions were protected from light and analyzed by using flow cytometry.

### 3.9. PP2A Activity Assay

PP2A activity assay was a PP2A immunoprecipitation phosphatase assay kit operated according to the manufacturer’s instruction. Briefly, a prepared protein aliquot was incubated with anti-PP2A subunit C-antibody and protein A agarose slurry at 4 °C for 2–3 h, and then phosphopeptide was added. Samples were recorded by Thermo Scientific Multiskan GO.

### 3.10. Western Blot Analysis

Cell extracts from MIA Paca2 cells were determined by BCA Protein Assay Kit (Thermo Scientific) according to the manufacturer’s instructions. Separate 20–30 μg protein by electrophoresis using Tris-Glycine gel and transfer to PVDF membranes. After transferring and blocking, the membrane was incubated with the primary antibody in TBS-T buffer containing 5% skim milk overnight at 4 °C. Then, the membrane was washed three times with TBS-T, and the secondary antibody was incubated. Chemiluminescence detection was performed using a Western blot detection kit (Milipore). Bax, Bcl-2, Caspase-3, PARP, AKT, p-AKT, ERK, and p-ERK were used for Western blot analysis. Internal controls were detected using Actin antibody.

### 3.11. In Vitro Metabolic Stability of PF-543, Derivative ***5***, and ***10***

Microsomal incubations were conducted in triplicate in 0.1 M potassium phosphate buffer (pH 7.4) in a clean 1.5 mL Eppendorf tube. To evaluate NADPH-dependent metabolism, PF-543, derivative **5**, **10**, or Verapamil (positive control) was incubated with pooled liver microsomes from human (HLM), dog (DLM), rat (RLM), and mouse (MLM) in the presence of NADPH regenerating system in a final volume of 100 mL. The final incubation mixtures contained 0.5 mg/mL HLM, 0.1 M potassium phosphate buffer (pH 7.4), NADPH regenerating system (1 mM NADPH, 10 mM MgCl_2_). HLM was added, and the mixture was pre-incubated at 37 °C in a shaking incubator at approximately 350 rpm for 5 min under a Thermo mixer (Eppendorf., Hamburg, Germany). Reactions were initiated by the addition of 1 mM NADPH and were then quenched by adding 40 µL of ice-cold acetonitrile containing 10 µM chlorpropamide (CPP) as an internal standard at 0 and 30 min. The incubation mixtures were then centrifuged at 15,000 g for 5 min at 4 °C. A 2 µL aliquot of the supernatant was directly injected into the LC-MS/MS system. The LC-MS/MS system consisted of the Nexera XR HPLC system (Shimadzu Co., Kyoto, Japan) coupled to the TSQ Vantage triple quadrupole mass spectrometer equipped with Xcalibur version 1.1.1 (Thermo Fisher Scientific Inc., Waltham, MA, USA).

### 3.12. Molecular Modeling Studies

Molecular modeling studies of FTY720 (Fingolimod) and compound **10** against the human I2PP2A/SET were performed using Schrödinger Suite 2021–2 (Schrödinger, LLC, http://www.schrodinger.com). The X-ray crystal structure was obtained from the Protein Data Bank (http://www.rcsb.org/pdb, PDB code: 2E50). The protein preparation was revised using Protein Preparation Wizard in Maestro v.12.8, and the receptor grid box was generated as 25 × 25 × 25 Å cubic size centered on complex ligands. All ligands were subjected to ligand preparation using the LigPrep module of Maestro. The force field was OPLS4. Using Epik to generate possible states at pH 7.0 ± 2. At most, 32 tautomers per ligand were generated while keeping other parameters as default. Flexible dockings were performed using the Glide v.9.1 program with a standard precision method.

### 3.13. Statistical Analysis

The data were presented as means ± SD. Non-parametric one-way ANOVA was applied to the data with heterogeneous variance. If the interaction was obtained as significant, Turkey’s post hoc procedure was performed to determine group-wise variation. The difference was considered significant if *p* < 0.05. Graph Pad Prizm 7 was used to analyze all the data.

## 4. Conclusions

We synthesized derivatives in which the benzenesulfonyl structure of PF-543 was transformed into a triazole structure. In compounds **1**–**5** with aromatic tails, the selectivity for SK1 was maintained, whereas in compounds **6**–**10** with aliphatic tails, the selectivity was reduced. These results show that the aromatic tail, such as in PF-543, is an important structure for the selectivity of SK1. In pancreatic cancer cells, compounds **6**–**10** containing a tail similar to that of FTY720 showed a relatively high cytotoxic effect. This finding shows that the aromatic tail structure, such as that present in PF-543, may not be favorable for anticancer activity. Compound **10**, which was found to inhibit both SK1 and SK2, reduced the S1P level to a degree similar to that of PF-543; however, unlike in the case of PF-543, the ceramide level increased, but the sphingosine level decreased. This difference in the sphingolipid level of compound **10** might be associated with the improvement of the low anticancer activity of PF-543. Compound **10** had the same tail structure as that of FTY720 and activated PP2A, but the docking analysis revealed that the tail of this compound bound to PP2A in a different direction depending on where the aliphatic tail was connected. The MS results of the compounds show that the tail structure of PF-543 may be responsible for its low MS, indicating that the triazole structure can increase the stability of PF-543. Our study demonstrated that the target of the novel PF-543 analogs should be identified through the simultaneous evaluation of their SK-inhibitory effect and PP2A activity.

## Figures and Tables

**Table 1:** In vitro stability study of compound **5**, **10**, and PF-543 compared with verapamil on human, dog, rat, and mouse liver microsomes.

Compounds	Human (%)	Dog (%)	Rat (%)	Mouse (%)
**5**	45.4	40.8	27.6	17.9
**10**	66.7	58.3	53.3	33.3
PF-543	5.8	2.9	4.7	7.5
Verapamil	9.1	-	-	-

## Data Availability

Not applicable.

## Supplementary Materials

The following are available online at https://www.mdpi.com/article/10.3390/molecules27103346/s1, Figure S1: Relative IC50 determination of Compound 5, Compound 10, PF-543, and FTY720.

## Sample Availability

Not available.
