# Supplementary material for: Synthesis of PP2A-Activating PF-543 Derivatives and Investigation of Their Inhibitory Effects on Pancreatic Cancer Cells
Source: Molecules. 2022 May 23;27(10):3346. doi: 10.3390/molecules27103346 (PMC9145885; doi:10.3390/molecules27103346)
Supplement: Supplementary file 1 [file molecules-27-03346-s001.zip › molecules-1741658-supplementary.pdf]

Supplementary Figure 1

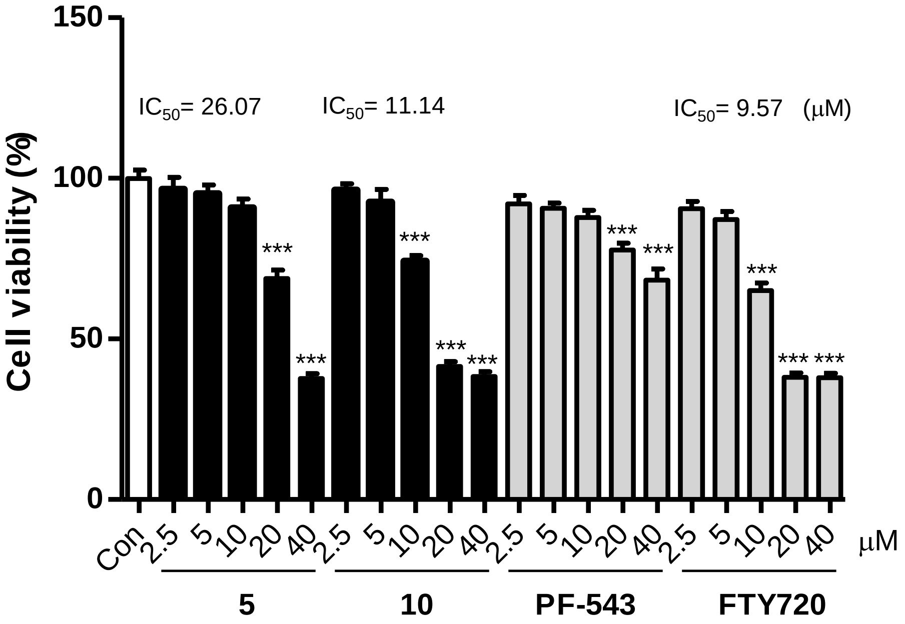

Supplementary Figure 1. Relative IC_{50} determination of Compound 5, Compound 10, PF-543, and FTY720. MIAPaCa2 cells were seeded in 96 well plates and treated with 2.5-40 μM of compounds for 24h. Cell viability was assessed using MTT assay, and Relative IC_{50} was determined using Graph pad prism software. The result is representative of three independent experiments and data are presented as mean ± SD. *** $p < 0.001$ compared with non-treated control group.
